# Ophthalmology

**Published:** 1901-08

**Authors:** Alvin A. Hubbell

**Affiliations:** Buffalo, N.Y.


### PROGRESS IN MEDICAL SCIENCE.

### Ophthalmology.

Conducted by ALVIN A. HUBBELL, M.D., Buffalo, N.Y.

### KRÖNLEIN'S OPERATION FOR REMOVAL OF TUMORS OF THE ORBITAL CAVITY.

Krönlein (*Ophthalmic Review*, January, 1901,) realising the difficulty of access to the apex of the orbital cavity, has devised an operation by which this may, in great measure, be overcome, and at the same time preserve the eyeball. His method may be summarised as follows: A skin incision is made in a curved line beginning at the ridge of bone—easily felt by the finger—which bounds anteriorly the temporal fossa, about 1 cm. above the orbital margin, curving forwards to have its middle point opposite the outer canthus, and terminating over the zygoma half way between the tragus and the external canthus. One advantage of the incision being thus curved, is that when the parts are replaced after the operation the soft parts are subjected to no tension, but fit quite readily. By means of a periosteum elevator, the periosteum covering the whole outer wall of the orbit, is now eased off its bed, and the point being carefully introduced into the inferior orbital fissure and the handle carried toward the nose, it is completely lifted off. This is not difficult, as it is only at the orbital margin and the sutures that the periosteum is at all firmly adherent. Three bone sections are now required—two horizontal and one oblique. The first divides the external angular process of the frontal bone close to its base, the next (oblique) runs from the deepest portion of the first behind the suture, which joins the malar and sphenoid, and ends in the spheno-maxillary fissure. This is best accomplished by means of a narrow sharp chisel, the orbital contents being kept well out of danger of injury meanwhile. The third passes through the frontal process of the malar, close to its root, and also ends in the spheno-maxillary fissure; it may be made either with a special small saw or with a sharp chisel. In order to have as much space as possible it is well to keep the two straight sections as far apart as may be, and much care should be taken to avoid any unnecessary splintering of the bones, especially during the chisel work.

For this reason the chisel ought to be both fine and very sharp, and the strokes of the hammer very light. The author devises a special instrument for this purpose, for whose characteristics reference to the original (p. 564) should be made, as well as for advice regarding special saws. The oblique incision must always be made before at least the second of the straight sections, else there is no firm support for the working of the chisel. The dimensions of the piece of bone thus loosened vary considerably with the shape of the particular orbit, but should be about 3 c.m. high and 2½ c.m. deep—that is, about an inch in depth and rather more than that in height. It is rolled outward without difficulty about its vertical axis along with the soft parts covering it, thus giving free access to the deeper parts of the orbit. The periosteum is now divided with blunt-pointed scissors from before backwards and turned aside, when the contents of the orbit are clearly seen. Further steps will, of course, depend on the condition of affairs then discovered, but generally the external rectus is divided close to the sclerotic insertion, and into both ends of it, and of any other muscles which requires division, stitches should be inserted for convenience of subsequent indentification and reposition. At the close of the operation the flap of bone and soft tissue overlying it is replaced, and held in position by a few stitches passing through the periosteum; a drain of tubing or iodoform gauze is left in for a few days; silk is usually employed for the skin wound.

---

### THE PATHOLOGY OF SERPENT ULCER OF THE CORNEA.

Dotsch, Dr. A., of the Jena Eye Clinic (*Die Ophthalmologische Klinik*, October 5, 1900; *Annals of Ophthalmology*, January, 1901.) This is a very interesting and important communication and contains a complete analysis of the cases of serpent ulcer seen in the Jena Eye Clinic during the years 1898 and 1899. The number of cases seen was 98. I think the chief point of interest lies in the results of the careful bacteriological examinations which were made in most of these cases. He has been able to confirm absolutely the conclusions of Uhthoff and Axenfeld, who, as we know, found in the majority of their cases the Frankel-Weichselbaum diplococcus; in other words, the pneumococcus. In 63 cases the pneumococcus was found 41 times and in 35 of these cases in pure culture. The history of an injury was noted in 69 cases. Inasmuch as this organism flourishes exceptionally outside of the human body it is doubtful whether it was introduced into the eye on the foreign body, but that the eye was subsequently infected by this organism, which we know, is not infrequently found in the mucous membrane adjacent to the eye. It would, seem, then, that pneumococcus ulcer is the proper designation for this variety of corneal trouble.

---

### ON THE ACTION OF SUPRARENAL EXTRACT.

Zimmerman, Stuttgart, (*La Clinique Ophthalmologique*, October 10, 1900; *Annals of Ophthalmology*, January, 1901.) Fresh solutions of dried suprarenal glands, Zimmerman states, may be introduced into the conjunctival cul-de-sac without producing any irritation. He has found that the conjunctival vessels contract and disappear from view first, but if the action of the material be continued sufficiently long, the scleral vessels will do the same. This he says, renders the drug of considerable service in inflammatory and congestive disturbances of the ocular structures. He has found that probably the most marked therapeutic action which may be noticed, is the ability of gland material to quiet certain intractable cases of glaucoma, particularly if it be used in connection with weak solutions of some of the older miotics. Darier's foimula, which contains three centigrams of chlorohydrate of pilocarpine and two centigrams of salicylate of eserine to ten grams of an equal part solution of dried suprarenal glands in water have given him the best results.

---

AMBLYOPIA FOLLOWING THE INTOXICATING USE OF JAMAICA GINGER.

EDWARD STIERIN (*Jour. of the American Med. Assn.*, January 5, 1901; *Amer. Jour. of Ophthalmology*, April, 1901,) finds eight recorded cases in American ophthalmic literature of blindness following the ingestion of ginger. His case was that of a man, 36 years of age, who had been drinking heavily. On sobering up in a place where he could not obtain liquor, he consumed one dozen ounce bottles of Jamaica ginger in one forenoon. About noon he fell into a deep stupor, from which he awoke about 3 p.m., totally blind. Treatment consisted in confinement to bed in a dark room; three hot foot baths were given during the night, and twenty grains each of calomel and compound jalap powder in divided doses; also two one-eighth grain doses of pilocarpine mur. hypodermatically. The pilocarpine and calomel were continued for two days, when they were discontinued and 20 grain doses of potassium iodide given. On the fifth day his vision was 20-30 in each eye. The lesion in these cases is believed to be a retrobulbar neuritis.

